# Atypical object exploration in infants at-risk for autism during the first year of lifer

**DOI:** 10.3389/fpsyg.2015.00798

**Published:** 2015-06-16

**Authors:** Maninderjit Kaur, Sudha M. Srinivasan, Anjana N. Bhat

**Affiliations:** ^1^Department of Physical Therapy, Biomechanics and Movement Science Program, University of Delaware, NewarkDE, USA; ^2^Adjunct Faculty, Physical Therapy Program, Department of Kinesiology, University of Connecticut, StorrsCT, USA; ^3^Behavioral Neuroscience Program, Department of Psychology, University of Delaware, NewarkDE, USA; ^4^Center for Health, Intervention, and Prevention, Department of Psychology, University of Connecticut, StorrsCT, USA

**Keywords:** autism, object exploration, infants, motor, social, communication, development

## Abstract

Autism Spectrum Disorder (ASD) is a neurodevelopmental disorder usually diagnosed by the end of the second year of life. Early signs of ASD within the first year of life are still unclear. The main purpose of the present study was to compare object exploration skills between infants at-risk for ASD and typically developing (TD) infants to determine early markers for autism within the first year of life. Sixteen at-risk infants and 16 TD infants were longitudinally followed from 6 to 15 months of age during an object exploration task involving three objects with distinct size, shape, and texture, i.e., a long rattle, a rigid circular ball, and a soft circular koosh ball. All sessions were videotaped for coding of manual exploration (grasping and dropping), oral exploration (mouthing), and visual exploration (looking). We also obtained follow-up outcomes using various developmental questionnaires at 18 months and email follow-up on developmental delays/ASD diagnosis after the infants’ second birthdays. Our results showed object-based differences in exploration patterns that extended across both groups. We also noticed context-dependent group differences for various exploratory behaviors across objects and ages. Specifically, at 6 months, at-risk infants showed less grasping of the rigid ball as well as less mouthing and greater looking at the rattle compared to TD infants. At 9 and 12 months, at-risk infants demonstrated significantly lower levels of purposeful dropping of all objects and greater looking at the rattle. Lastly, at 15 months, at-risk infants showed persistent mouthing of the rigid ball and rattle compared to TD infants. In addition, 10 out of 16 at-risk infants developed various motor, social, and language delays or ASD diagnosis at follow-up. Taken together, early context-dependent delays/abnormalities in object exploration could be markers for future developmental delays in infants at-risk for autism. Moreover, promoting early object experiences through socially embedded, free and structured play could have significant implications for multisystem development including perceptuo-motor, social communication, and cognitive development in at-risk infants.

## Introduction

Autism Spectrum Disorder (ASD) is a neurodevelopmental disorder characterized by impairments in social communication development such as lack of reciprocity during social interactions, reduced use of communicative gestures, and a complete lack of or delay in language development, as well as by the presence of restricted and repetitive behaviors such as hand flapping and preoccupation with objects (Mitchell et al., 2006; Sullivan et al., 2007; Eigsti et al., 2011; Leekam et al., 2011; American Psychiatric Association, 2013). The current prevalence of ASD is 1 in 68 children (Centers for Disease Control and Prevention, 2014) with diagnostic confirmation usually by the second year of life (Robins et al., 2001; Shattuck et al., 2009). Early detection in the second year of life gives families access to appropriate behavioral interventions and is known to improve future outcomes (Osterling and Dawson, 1994; Rogers, 1998; Fein et al., 2013). Early detection studies have typically reported retrospective data on infants who later developed ASD as well as prospective data in infant siblings of children with ASD or AU sibs. Although diagnostic features of autism are within the social communication domains, some of the early signs of autism within the first year have been observed in the perceptuo-motor domains (Teitelbaum et al., 1998; Gernsbacher et al., 2008; Ozonoff et al., 2008b). Retrospective reports suggested that early signs of autism include motor delays (Teitelbaum et al., 1998; Gernsbacher et al., 2008; Ozonoff et al., 2008b) as well as excessive visual exploration of objects (Maestro et al., 2002, 2005; Bhat et al., 2010). However, recent prospective studies have identified subtle atypicalities specific to autism as early as the first year of life. During free exploration of objects, AU sibs showed reduced mouthing and grasping as well as excessive looking at 6 and 9 months of age (Koterba et al., 2012; Libertus et al., 2014). Therefore, the present study builds on the current literature by conducting a prospective longitudinal study comparing object exploration skills between at-risk infants and age-matched, typically developing (TD) infants from 6 to 15 months of age with developmental questionnaire follow-up at 18 months.

Object exploration refers to infants’ exploration of toys and objects using oral (i.e., mouthing), manual (i.e., grasping, fingering, shaking, banging, rotating), and visual (i.e., looking) modalities (Ruff, 1984; Palmer, 1989). In order to explore objects in different ways, infants require substantial fine motor and gross motor skills. For example, manual modes of exploration such as fingering, shaking, transferring, and rotating objects require considerable hand and finger control (Needham et al., 2002; Barrett et al., 2008). Similarly, good trunk control is critical for proficient use of arms while exploring objects (Rochat and Goubet, 1995; Lobo and Galloway, 2008). In fact three weeks of enhanced postural training led to improved reaching, mouthing, and fingering of objects in 2- to 5-month-old TD infants (Lobo and Galloway, 2008). Moreover, object exploration skills have implications for other forms of development such as perceptual (Needham, 2000; Bhat and Galloway, 2006; Lobo and Galloway, 2008; Koterba et al., 2012), social communication (Meltzoff, 1995; Fagan and Iverson, 2007; Iverson et al., 2007), and cognitive development (Caruso, 1993; Bourgeois et al., 2005; Fontenelle et al., 2007). In terms of perceptual development, infants learn various object properties such as texture, shape, size, color, and sound while exploring objects (Ruff, 1984, 1986; Palmer, 1989; Rochat, 1989). For example, 3- to 4-month-old infants who spent more time exploring objects had better perception of object properties such as the boundaries of two closely placed objects compared to infants who spent less time exploring objects (Needham, 2000). Infants’ experience with objects improves their object knowledge and directly affects their performance in various cognitive tasks (Caruso, 1993; Bourgeois et al., 2005). When infants were asked to retrieve a toy from a container, their success directly correlated with their object exploration abilities. Specifically, infants who spent majority of their time exploring objects were more successful and used different strategies to retrieve the toy from the container (Caruso, 1993). Lastly, object play promotes both non-verbal communication skills such as showing and pointing (Iverson and Goldin-Meadow, 2005) as well as verbal communication skills such as vocalizations produced while exploring objects (Fagan and Iverson, 2007; Iverson et al., 2007) and labeling of objects (Baldwin and Markman, 1989). Specifically, rhythmic shaking of the rattle was closely related to babble onset in 4- to 9-month-old infants (Iverson et al., 2007) and mouthing of objects was closely associated with consonant production in 6- to 9-month-olds (Fagan and Iverson, 2007). Overall, object exploration could be a valuable paradigm to examine various forms of development in the first year of life. Next, we will be discussing the current literature on developmental trends in object exploration skills in TD infants and infants at-risk for autism.

Infants show substantial improvements in object exploration skills from birth to the end of the first year of life. Several factors including advancing age, improvements in motor skills, novelty of objects, as well as object properties influence infants’ exploratory behaviors. Even newborn infants show differential oral and manual responses to objects of varying texture and rigidity (Rochat, 1987). However, active object exploration emerges around 3- to 6-months of age with the onset of reaching and grasping (Ruff, 1984; Rochat, 1989; Lobo and Galloway, 2008). At 6 months of age, infants spent the majority of their time mouthing and grasping objects and this sharply declined around 12- to 15-months of age with concurrent improvements in complex manual exploratory behaviors such as fingering, transferring, and rotating objects (Belsky and Most, 1981; Ruff, 1984). These improved fine motor skills may allow infants to perceive additional structural details of objects. In terms of visual exploration, early on, infants engaged in looking behaviors in isolation; however, older infants looked at objects while simultaneously fingering, turning, or rotating them (Ruff, 1986; Ruff et al., 1992). Looking accompanied with manual exploration provides infants with greater information about object properties than looking alone. Moreover, older infants showed preferential looking toward novel objects than familiar objects (Ruff, 1986). In the current study, we were interested in comparing the developmental trajectories for visual, oral, and manual exploratory behaviors in TD and at-risk infants over the first 15 months of life.

Exploratory behaviors are also influenced by object properties including size, shape, texture, and weight of objects, often called object affordances or natural opportunities for actions on objects (Newell et al., 1989, 1993; van Hof et al., 2002; Bourgeois et al., 2005; Barrett et al., 2008; Corbetta and Snapp-Childs, 2009; Libertus et al., 2013). For example, infants showed greater grasping of smaller objects whereas they looked more at larger, perceivable objects (Rochat, 1989). In addition, 9- to 12-month-old infants explored object properties such as shape, size, and texture by rotating, fingering, and transferring objects, whereas they explored properties such as weight, sound, and rigidity by banging and shaking objects (Ruff, 1984). Infants’ grasping patterns depended on object size such that smaller objects were grasped unimanually and larger objects were grasped bimanually. Similarly, infants squeezed non-rigid objects more compared to rigid objects (Newell et al., 1989, 1993; Barrett et al., 2008). Given the interactions between object affordances and exploratory strategies of TD infants, we were interested in examining whether at-risk infants suitably and flexibly adapted their exploratory strategies to different object affordances over the first 2 years of life.

Unusual object exploration in the first year of life has been reported in retrospective studies in infants who later developed ASD as well as prospective studies comparing infants at-risk for autism and TD infants. Some abnormalities include excessive mouthing (Baranek, 1999; Bhat et al., 2009; Koterba et al., 2012), excessive visual fixation (Maestro et al., 2002; Zwaigenbaum et al., 2005; Bhat et al., 2010; Koterba et al., 2012; Chawarska et al., 2013), and repetitive use of objects (Ozonoff et al., 2008a). During the first year of life, AU sibs showed distinct mouthing patterns such as less mouthing of objects as early as 6 months (Bhat et al., 2009; Koterba et al., 2012). In contrast, excessive mouthing was reported at 9- and 12-months in infants who later developed ASD (Baranek, 1999). This developmental trajectory for mouthing differs compared to TD infants who predominantly use oral exploration at 6 months but transition to more advanced forms of manual exploration at 9 months with a concurrent decrease in oral exploration (Belsky and Most, 1981; Ruff, 1984). In terms of visual exploration in the first year of life, there is converging evidence from retrospective and prospective studies that infants at-risk for autism show greater visual fixation on objects (Zwaigenbaum et al., 2005; Koterba et al., 2012) and less attention toward social stimuli including caregivers and experimenters compared to TD infants (Maestro et al., 2002; Bhat et al., 2010; Chawarska et al., 2013). These unusual visual attention patterns continue from infancy into early childhood (Swettenham et al., 1998; Mottron et al., 2007; Shic et al., 2011; Chawarska et al., 2012). Lastly, several studies have also reported repetitive use of objects including less functional play between 9 and 12 months (Baranek et al., 2005) and excessive spinning of objects at 12 months (Ozonoff et al., 2008a) in AU sibs and infants who eventually developed ASD. Overall, there is considerable evidence supporting the presence of delayed and atypical object exploration skills in at-risk infants within the first year.

In spite of the unequivocal nature of the evidence supporting the early atypical nature of object exploration in infants at-risk for autism, there are several gaps in this literature. Specifically, studies have restricted their examination of at-risk infants to specific ages or to specific types of exploration. For instance, Ozonoff et al. (2008a) compared the object exploratory skills of at-risk infants who eventually developed ASD with those of TD infants at 12 months of age. Similarly, other studies restricted their examination of object exploration skills of infants to only two time-points within the first year (Baranek et al., 2005; Bhat et al., 2009; Koterba et al., 2012; Libertus et al., 2014). Along the same lines, the majority of the studies have evaluated a single type of skill such as manual, oral, or visual exploration in isolation (Maestro et al., 2002; Baranek et al., 2005; Bhat et al., 2010; Libertus et al., 2014). A comprehensive understanding of the developmental trajectory of object exploration skills in at-risk infants would require studying different forms of exploration in conjunction over the course of development. Moreover, given that exploratory strategies employed by infants are influenced by object affordances, it would be critical to consider object properties while studying exploratory behaviors. For instance, group differences in object exploration may be highly context-dependent; in other words, they may be revealed only during specific types of exploration involving specific objects at specific time points in development. Therefore, it would be important to assess different forms of exploration over the course of development with objects providing a variety of affordances. In the current longitudinal study, we aimed to concurrently and systematically examine different forms of object exploration including oral, visual, and manual behaviors as infants explored three objects of varying sizes, shapes, and textures, namely a rattle, a rigid ball, and a koosh ball from 6 to 15 months of age. We think that this design will allow us to better understand the context-dependency of group differences between TD and at-risk infants. This in turn will have significant implications for screening and identification of delays in at-risk infants within the first year of life. In the present study, we compared the manual (grasping and dropping), oral (mouthing), and visual (looking) exploration skills of TD and at-risk infants as they explored three different objects – a rattle, a rigid ball, and a koosh ball at 6, 9, 12, and 15 months of age.

Our first aim was to assess object-related differences or differences in how infants’ explored the specific object affordances. We hypothesized that both TD and at-risk infants would perceive object affordances and adapt their actions on objects accordingly. For example, infants would demonstrate greater grasping and mouthing of the easily graspable rattle, greater dropping of the sounding rigid ball, and greater looking at the novel koosh ball. Our second aim was to examine group differences in object exploration skills between at-risk and TD infants from 6 to 15 months of age. We hypothesized that at-risk infants would show delays in age-appropriate exploration of objects compared to TD infants. Specifically, they would demonstrate context-dependent differences such as less grasping and mouthing at an early age, as well as less purposeful dropping, greater looking, and persistent mouthing at an older age. Lastly, we were interested in examining any shifts/delays in the developmental trajectories for different forms of exploration in at-risk infants compared to TD infants. We hypothesized that TD infants would replace immature exploratory behaviors such as mouthing with more advanced forms of information-gathering behaviors such as grasping and dropping from 6 to 15 months of age. In contrast, we expected at-risk infants to show a delayed developmental transition from immature to more advanced forms of object exploration.

## Materials and Methods

### Participants

Sixteen infants at-risk for autism (14 AU sibs and two preterm infants who later developed ASD) and 16 TD full term infants with no significant birth history or family history of ASD were observed over four visits at 6, 9, 12, and 15 months of age (see **Table 1**) within the object exploration paradigm. In terms of socioeconomic status, all families belonged to the upper–middle or upper class (Hollingshead, 1975, see details in Table 1). Participants were recruited through local day care centers, autism service providers such as clinics and schools, web postings, and word of mouth. We excluded infants with significant birth history including low birth weight, head injury, birth trauma, any known genetic disorder, hearing or vision impairment, or any orthopedic or other medical diagnoses that could affect participation. The older siblings of all 14 AU sibs met diagnostic criteria for ASD based on the Autism Diagnostic Interview-Revised (ADI-R; Lord et al., 1994), expert clinical judgment, and/or medical records. Two preterm twins were enrolled in the study with no specific diagnoses as our research protocol was broader and included multiple at-risk populations. Both preterm infants were diagnosed with ASD in the second year of life based on the aforementioned criteria; hence, we have included their data within the group of at-risk infants. All parents signed the formal parental permission form approved by the University of Connecticut’s Review Board before participating in the study.

**Table 1:** Participant characteristics.

Group	*n*, Gender F:M	Ethnicity C, AA/M	SES Mean ± SD	Age in months (Mean ± SD)			
				6	9	12	15
Typically developing (TD)	16, 6:10	15 C, 1 AA	55.32 ± 9.22	6.93 ± 0.60	10.02 ± 0.46	12.98 ± 0.69	15.86 ± 0.46
At-risk	16, 3:13	15 C, 1 M	52.03 ± 12.66	6.83 ± 0.60	9.70 ± 0.55	12.97 ± 0.89	15.62 ± 1.13
*p*s	ns	ns	ns	ns	ns	ns	ns

N, total number of participants in each group; F:M, total number of females and males in each group; SES, socioeconomic status; C, Caucasian; AA, African American; M, Mixed; ns, *p*-values not significant (i.e., *p*s > 0.05).

### Future Outcomes of At-Risk Infants

We obtained developmental outcomes for infants in both groups at 18 months using parent questionnaires, the Ages and Stages Questionnaire-third edition (ASQ-3; Squires et al., 1999) and the Modified Checklist for Autism in Toddlers (M-CHAT; Robins et al., 1999, 2001). The ASQ-3 has multiple developmental domains of personal–social, communication, gross and fine motor, and problem solving/cognitive abilities (Squires et al., 1999). A developmental delay was defined as a total score ≤1SD below the mean standard score. M-CHAT is a 23-item yes/no checklist for the screening of ASD and a failure on any three items or two critical items indicates higher risk to develop ASD (Robins et al., 1999, 2001). Fifteen out of 16 parents of TD infants and 14 out of 16 parents of at-risk infants filled out the 18-month questionnaires (see details in **Table 2**). None of the TD infants reported significant delays on the ASQ-3 and M-CHAT. Among at-risk infants, eight reported delays on one or more domains of the ASQ-3 and six failed on the M-CHAT (see **Table 2**). We also conducted follow-up email inquiries with parents after the toddler’s second birthday regarding any developmental delays, diagnoses, and services received. None of the TD infants received developmental diagnoses at 2 years whereas eight at-risk infants developed delays/ASD diagnosis, specifically, five at-risk infants had language delays and three received an ASD diagnosis. In terms of services received, six of the eight at-risk infants with formal diagnoses/delays were receiving early intervention services based on parent emails (see **Table 2**). Taken together, 10 AU sibs received poor outcomes; of those six AU sibs failed on the M-CHAT, demonstrated delays on the ASQ-3 and parents reported developmental delays/ASD diagnosis during email follow-up. The parents of the preterm twins did not complete the 18-month questionnaire but reported ASD diagnosis during email follow-up. Lastly, two other AU sibs reported multiple delays on the ASQ-3 but did not follow-up via email. These findings clearly distinguish the group of at-risk infants from the TD group; but due to small sample sizes we will not be distinguishing the performance of toddlers who developed future delays/diagnoses from those who did not report any delays. However, individual data have been reported for the at-risk group in the results section (see **Figures 7A–D**).

**Table 2:** Future outcomes of at-risk infants.

Group	ASQ-3 (18 months)			M-CHAT (18 months)	Emails – ASD Diagnosis/ Delays	Emails –Receiving Services
	Personal social and communication	Gross and fine motor	Problem solving
At-risk	6/14	3/14	2/14	6/14	8/14	6/14

### Experimental Set Up

**FIGURE 1:**
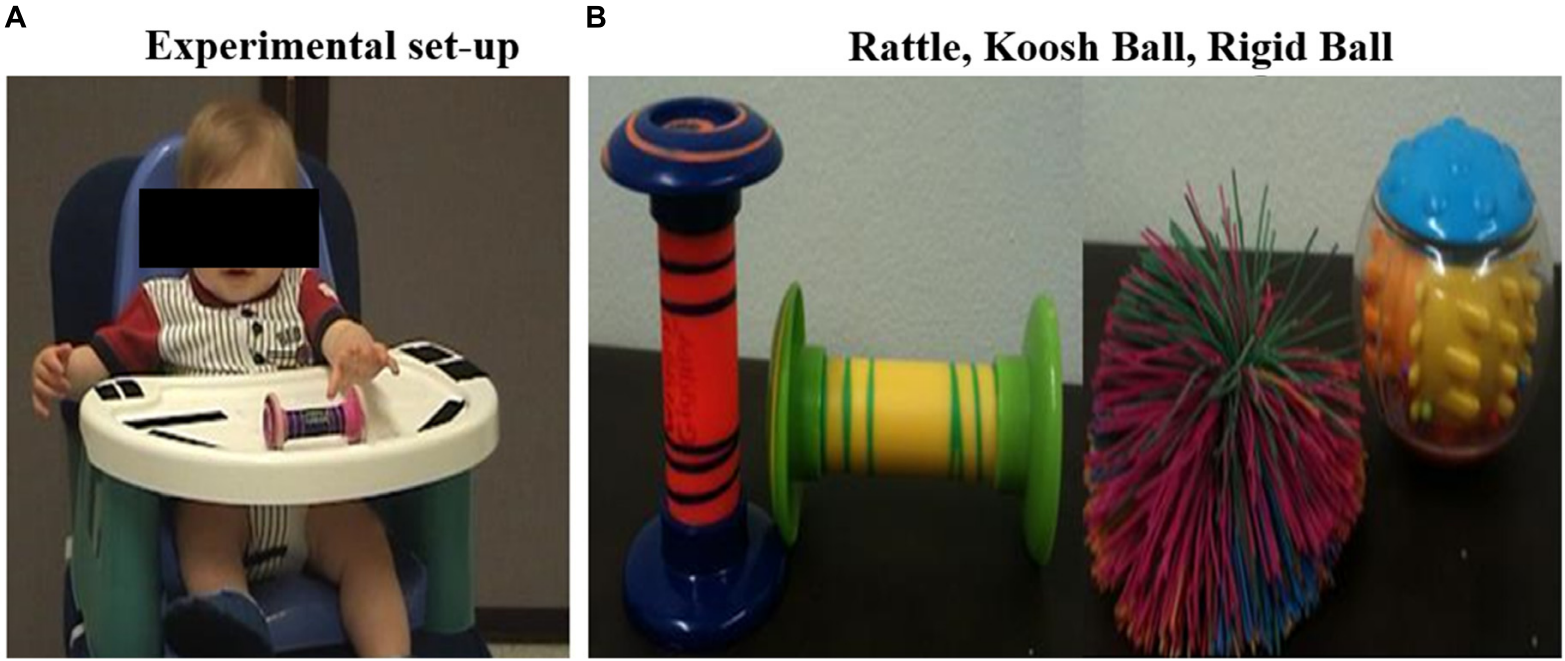
**The experimental set up with an infant sitting in a booster seat **(A)** and the objects presented – a rattle, a koosh ball, and a rigid ball (B)**.

Infants were seated upright in a booster seat with the tester on one side (see **Figure 1A**). A long rattle, circular rigid ball, and circular koosh ball were presented one at a time at the center of the tabletop within the infant’s reach (see **Figure 1B**). These objects were chosen as they varied in size, shape, and texture and hence afforded different types of exploratory behaviors. For example, the rattle afforded shaking and grasping, the sounding rigid ball afforded dropping/throwing, and the novel koosh ball afforded looking and holding. The presentation was in a fixed order – the rattle, the rigid ball, and then the koosh ball. The tester demonstrated the properties of objects, i.e., shaking of the rattle and the rigid ball or pulling strings of the koosh ball before presenting the object. The infant was allowed to freely explore the object for about one minute. If the object was dropped on the floor, it was presented again to the infant. Each session was videotaped for further coding. In terms of missing data, TD infants missed their visits as follows – 0/16 at 6 months, 2/16 at 9 months, 0/16 at 12 months, and 4/16 at 15 months. Similarly at-risk infants missed visits as follows - 3/16 at 6 months, 1/16 at 9 months, 0/16 at 12 months, and 1/16 at 15 months. The visits were missing due to later recruitment, illnesses, and/or scheduling conflicts.

### Behavioral Coding

A custom coding scheme was used to code the duration of each exploratory behavior using frame-by-frame analysis. Grasping was any form of manual contact with the object including higher level behaviors such as holding, shaking, banging, and fingering but excluding low level behaviors such as touching. Dropping was coded when the object was out of the infant’s hand including instances of accidental slips and purposeful drops. Mouthing was coded when the object was in contact with the infant’s mouth; this required infants to grasp the object and bring it to their mouth. Looking was visual fixation on the object when it was on the table or grasped. The percent duration of each behavior was calculated for each object presentation. Intra-class correlations (ICCs) were used to determine intra- and inter-rater reliability using 36 min of the dataset for each behavior. Intra- and inter-rater reliability scores were greater than 85% for the various exploratory behaviors based on ICC coefficients (grasping ≥0.88, dropping ≥0.93, mouthing ≥0.99, and looking ≥0.95).

### Statistical Analysis

We conducted a single Pillai’s Trace Multivariate Analysis of Variance (ANOVA) with behavior (grasping, dropping, mouthing, looking), age (6, 9, 12, 15 months) and object (rattle, rigid, koosh) as within-subjects factors and group (TD group, at-risk group) as the between-subjects factor. As mentioned previously, an important aim of our study was to compare group differences in object exploration skills between at-risk and TD infants. Hence, we conducted two types of planned comparisons: (a) group differences at each age were examined using independent *t*-tests and (b) developmental changes in exploratory behaviors were examined using dependent *t*-tests within each group. We will report group differences as early (at 6 months), mid (at 9 and 12 months), and late (at 15 months) differences. Similarly, we will report on developmental changes in exploratory behaviors as early (from 6 to 9 months), mid (from 9 to 12 months), and late (from 12 to 15 months) changes. We considered *p* ≤ 0.05 as significant for all the comparisons. The missing values were replaced with the average of the group for any given visit.

## Results

### Object-Based Differences in Exploratory Behaviors in TD and At-Risk Infants

**FIGURE 2:**
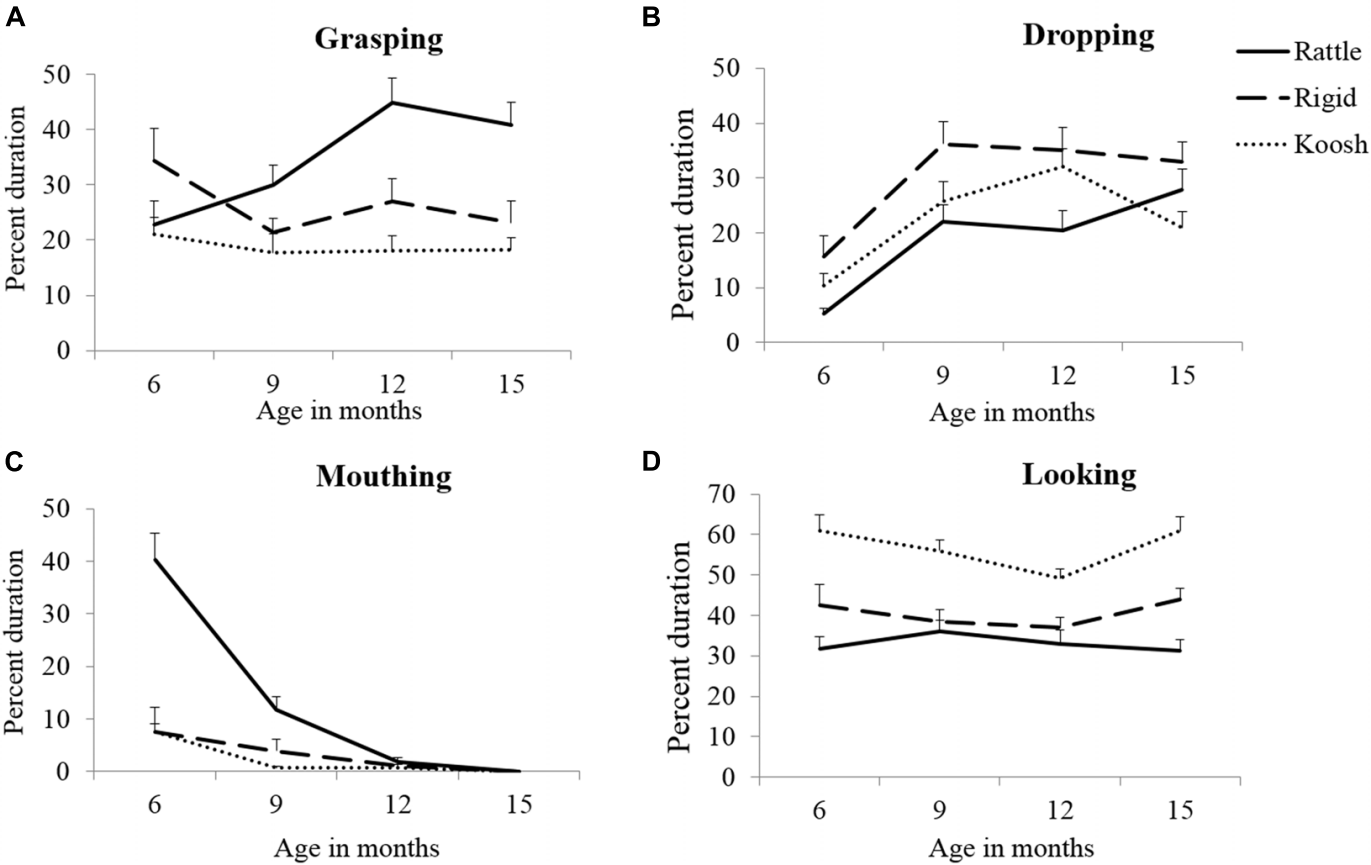
**Object-based differences in grasping **(A)**, dropping **(B)**, mouthing **(C)**, and looking **(D)** in typically developing infants**.

Both TD and at-risk infants clearly demonstrated differential exploration of the rattle, rigid ball, and the koosh ball suggesting that both groups perceived object affordances. Specifically, infants demonstrated greater grasping of the rattle (see **Figure 2A**) compared to other objects. There was more dropping of the sounding rigid ball compared to the other objects (see **Figure 2B**). Similarly, both TD and at-risk infants demonstrated greater mouthing of the rattle compared to the other objects (see **Figure 2C**). Lastly, there was more time spent looking at the koosh ball compared to the rattle and rigid ball (see **Figure 2D**). In terms of individual data, 12–16 out of the 16 TD infants and 9–6 out of the 16 at-risk infants followed their respective group trends.

### Group Differences and Differences in Development of Object Exploration in TD and At-Risk Infants

The multivariate analysis showed a significant main effect of behavior [Pillai’s Trace = 0.96, *F*(3,28) = 208.92, *p* < 0.05, $\eta_{p}^{2}$ = 0.96] and several interactions with behavior as a factor, including, behavior × object [Pillai’s Trace = 0.94, *F*(6,25) = 65.76, *p* < 0.05, $\eta_{p}^{2}$ = 0.94], behavior × age [Pillai’s Trace = 0.80, *F*(9,22) = 9.62, *p* < 0.05, $\eta_{p}^{2}$ = 0.80], behavior × age × group [Pillai’s Trace = 0.65, *F*(9,22) = 4.61, *p* < 0.05, $\eta_{p}^{2}$ = 0.65], and behavior × object × age [Pillai’s Trace = 0.86, *F*(18,13) = 4.44, *p* < 0.05, $\eta_{p}^{2}$ = 0.86]. Hence, we conducted separate ANOVAs for each behavior. Based on our planned comparisons, we analyzed the three-way or two-way interactions for each of the four exploratory behaviors to report group differences at each age and developmental changes in each group.

#### Grasping

The ANOVA for duration of grasping showed significant main effects of object [*F*(2,30) = 66.23, *p* < 0.05, $\eta_{p}^{2}$ = 0.69] and age [*F*(3,30) = 4.54, *p* < 0.05, $\eta_{p}^{2}$ = 0.13], as well as interaction effects of object × age [*F*(6,30) = 6.01, *p* < 0.05, $\eta_{p}^{2}$ = 0.17] and object × age × group [*F*(6,30) = 4.60, *p* < 0.05, $\eta_{p}^{2}$ = 0.13].

##### Group differences for grasping

**FIGURE 3:**
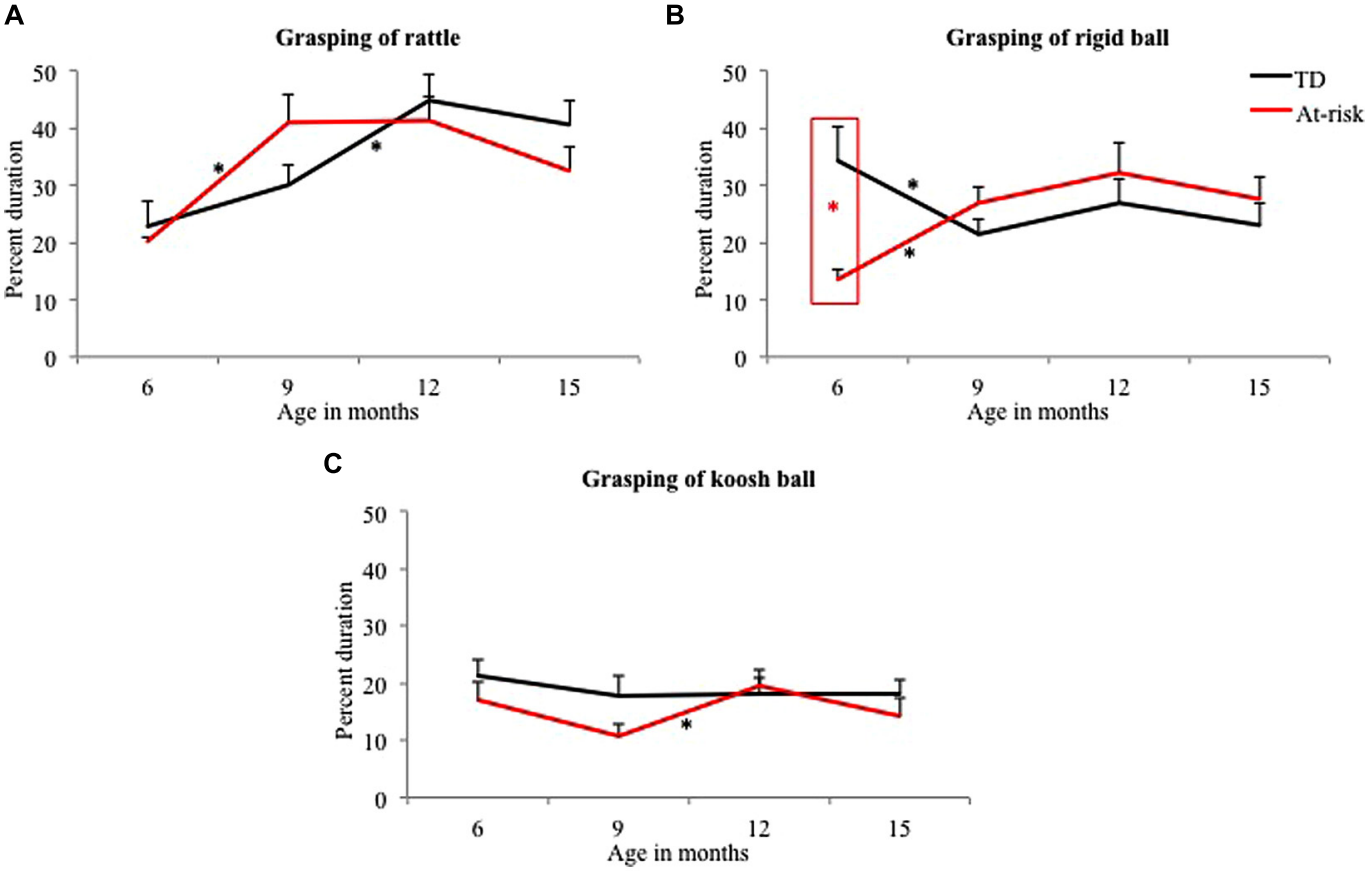
**Group differences and developmental trends for grasping of rattle **(A)**, rigid ball **(B)**, and koosh ball **(C)** in typically developing and at-risk infants.** ^∗^indicates *p* < 0.05. The red ^∗^ within a red box indicates a group difference and black ^∗^ indicates a developmental change between the two ages for the group indicated.

Early group differences were observed for grasping with at-risk infants showing less grasping of the rigid ball at 6 months compared to TD infants (see **Figure 3B**; **Table 3**). No other group differences were observed for grasping behaviors.

**Table 3:** *P-*values for group differences in object exploration between TD and at-risk infants.

Behavior	Age (in months)			
	6	9	12	15
Grasping	<0.01 (RB)	ns	ns	ns
Dropping	0.03 (A)	<0.01 (A)	<0.01 (A)	ns
Mouthing	0.02 (R)	ns	ns	<0.01 (R) 0.05 (RB)
Looking	0.02 (R)	ns	<0.01 (KB)	ns

R, Rattle; RB, Rigid Ball; KB, Koosh Ball; A, All objects; ns, *p-*values not significant.

##### Developmental changes in grasping

In terms of *early changes,* TD infants showed reduced grasping of the rigid ball between 6 and 9 months (see **Figure 3B**; **Table 4**) with no clear changes for the rattle and koosh ball. In contrast, at-risk infants significantly increased the grasping of the rattle and the rigid ball with no changes for the koosh ball (see **Figures 3A,B**; **Table 4**). In terms of *mid changes*, TD infants increased grasping of the rattle and at-risk infants increased grasping of the koosh ball between 9 and 12 months (see **Figures 3A,C**; **Table 4**). No late changes were observed for both groups.

**Table 4:** *P-*values for developmental changes in object exploration in TD and at-risk infants.

Behavior	Early (6–9 months)		Mid (9–12 months)		Late (12–15 months)	
	TD	At-risk	TD	At-risk	TD	At-risk
Grasping	0.02 (RB)	<0.01 (R,RB)	0.02 (R)	0.03 (KB)	ns	ns
Dropping	<0.01 (A)	ns	ns	ns	ns	<0.01 (A)
Mouthing	<0.01 (R)	<0.01 (R) 0.02 (KB)	<0.01 (R)	ns	0.05 (R)	ns
Looking	ns	ns	ns	ns	<0.01 (KB)	ns

R, Rattle; RB, Rigid Ball; KB, Koosh Ball; A, All objects; ns, *p-*values not significant.

#### Dropping

The ANOVA for duration of dropping indicated main effects of object [*F*(2,30) = 19.36, *p* < 0.05, $\eta_{p}^{2}$ = 0.39] and age [*F*(3,30) = 12.92, *p* < 0.05, $\eta_{p}^{2}$ = 0.30], as well as an age × group interaction [*F*(3,30) = 5.81, *p* < 0.05, $\eta_{p}^{2}$ = 0.16].

##### Group differences for dropping

**FIGURE 4:**
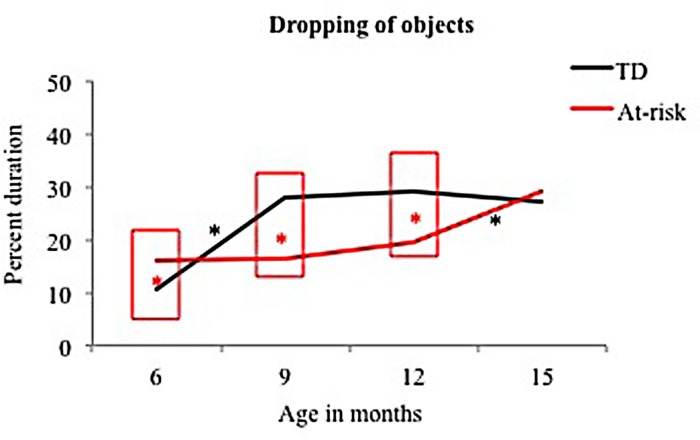
**Group differences and developmental trends for dropping of objects in typically developing and at-risk infants.** ^∗^indicates a *p* < 0.05. The red ^∗^ within a red box indicates a group difference and black ^∗^ indicates a developmental change between the two ages for the group indicated.

Significant early and mid group differences emerged for dropping behaviors. Specifically, at-risk infants engaged in greater dropping of objects at 6 months but lower levels of dropping at 9 and 12 months compared to TD infants (see **Figure 4**; **Table 3**).

##### Development changes in dropping

Typically developing infants showed an early increase in dropping of objects from 6 to 9 months whereas at-risk infants showed a delayed increase in dropping of objects from 12 to 15 months (see **Figure 4**; **Table 4**).

#### Mouthing

The ANOVA for mouthing duration indicated significant main effects of object [*F*(2,30) = 29.42, *p* < 0.05, $\eta_{p}^{2}$ = 0.50] and age [*F*(3,30) = 40.36, *p* < 0.05, $\eta_{p}^{2}$ = 0.57] as well as interaction effects of object × age [*F*(6,30) = 17.81, *p* < 0.05, $\eta_{p}^{2}$ = 0.37] and object × age × group [*F*(6,30) = 4.47, *p* < 0.05, $\eta_{p}^{2}$ = 0.13].

##### Group differences for mouthing

**FIGURE 5:**
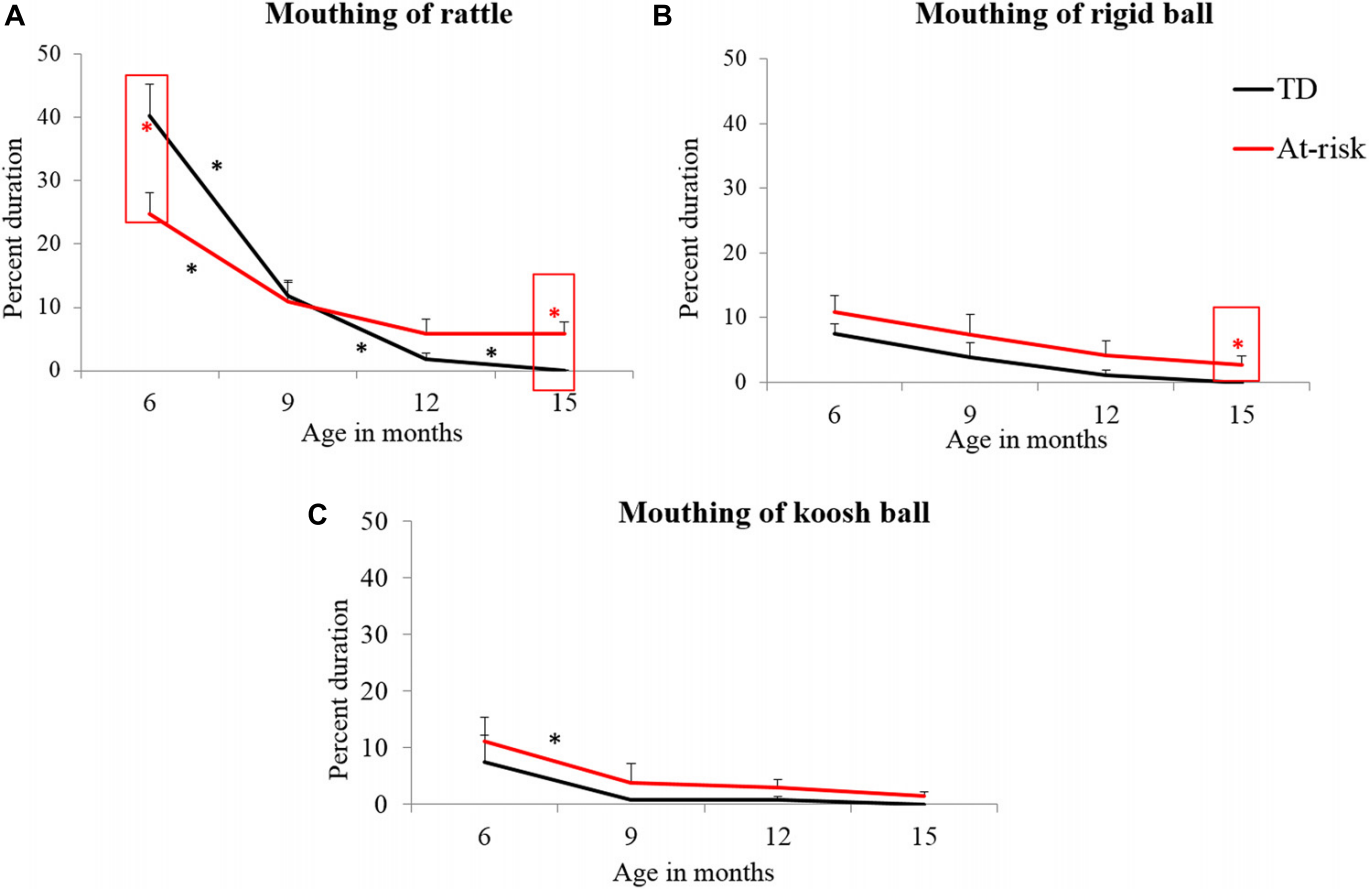
**Group differences and developmental trends for mouthing of rattle **(A)**, rigid ball **(B)**, and koosh ball **(C)** in typically developing and at-risk infants.** ^∗^indicates a *p* < 0.05. The red ^∗^ within a red box indicates a group difference and black ^∗^ indicates a developmental change between the two ages for the group indicated.

Significant early and late group differences were seen for mouthing behaviors. At-risk infants showed less mouthing of the rattle at 6 months (see **Figure 5A**; **Table 3**) and greater mouthing of the rattle and rigid ball at 15 months of age compared to TD infants (see **Figures 5A,B**; **Table 3**).

##### Development changes in mouthing

In terms of *early changes*, both TD and at-risk infants significantly decreased mouthing of the rattle from 6 to 9 months (see **Figure 5A**; **Table 4**). At-risk infants also decreased mouthing of the koosh ball from 6 to 9 months (see **Figure 5C**; **Table 4**). In terms of *mid and late changes*, TD infants continued to reduce mouthing of the rattle whereas no significant reductions in mouthing were observed in at-risk infants (see **Figure 5A**; **Table 4**).

#### Looking

The ANOVA for looking duration showed a main effect of object [*F*(2,30) = 88.11, *p* < 0.05, $\eta_{p}^{2}$ = 0.74] and a significant object × age × group interaction [*F*(6,30) = 3.20, *p* < 0.05, $\eta_{p}^{2}$ = 0.10].

##### Group differences for looking

**FIGURE 6:**
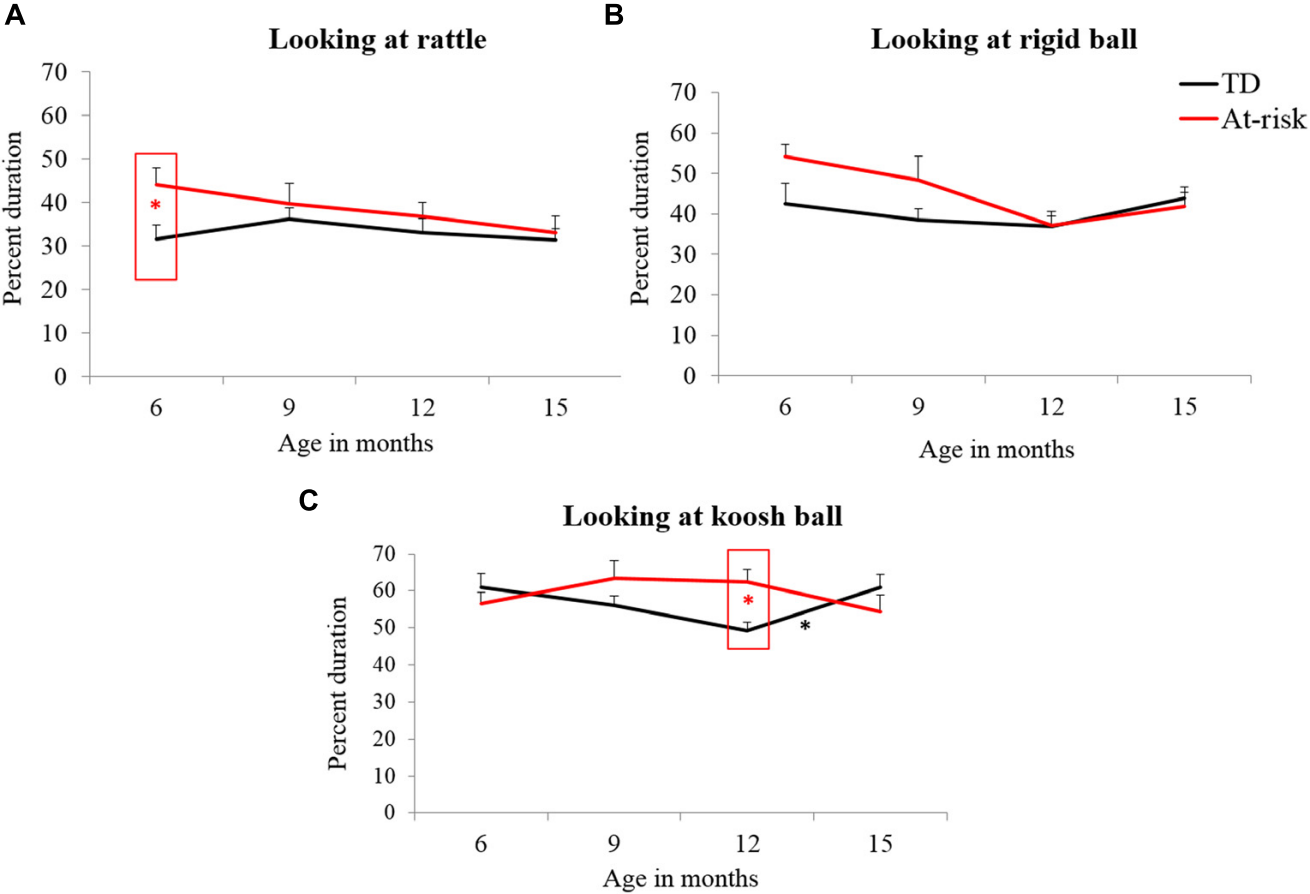
**Group differences and developmental trends for looking at rattle **(A)**, rigid ball **(B)**, and koosh ball **(C)** in typically developing and at-risk infants.** ^∗^indicates a *p* < 0.05. The red ^∗^ within a red box indicates a group difference and black ^∗^ indicates a developmental change between the two ages for the group indicated.

Significant early and mid group differences were observed for looking behaviors. At-risk infants spent greater time looking at the rattle at 6 months (see **Figure 6A**; **Table 3**) and at the koosh ball at 12 months (see **Figure 6C**; **Table 3**) compared to TD infants.

##### Developmental changes in looking

In terms of early, mid, and late changes, both groups showed no major changes in looking patterns (see **Figures 6A–C**; **Table 4**) except increased looking at the koosh ball in TD infants between 12 and 15 months (see **Figure 6C**; **Table 4**).

**FIGURE 7:**
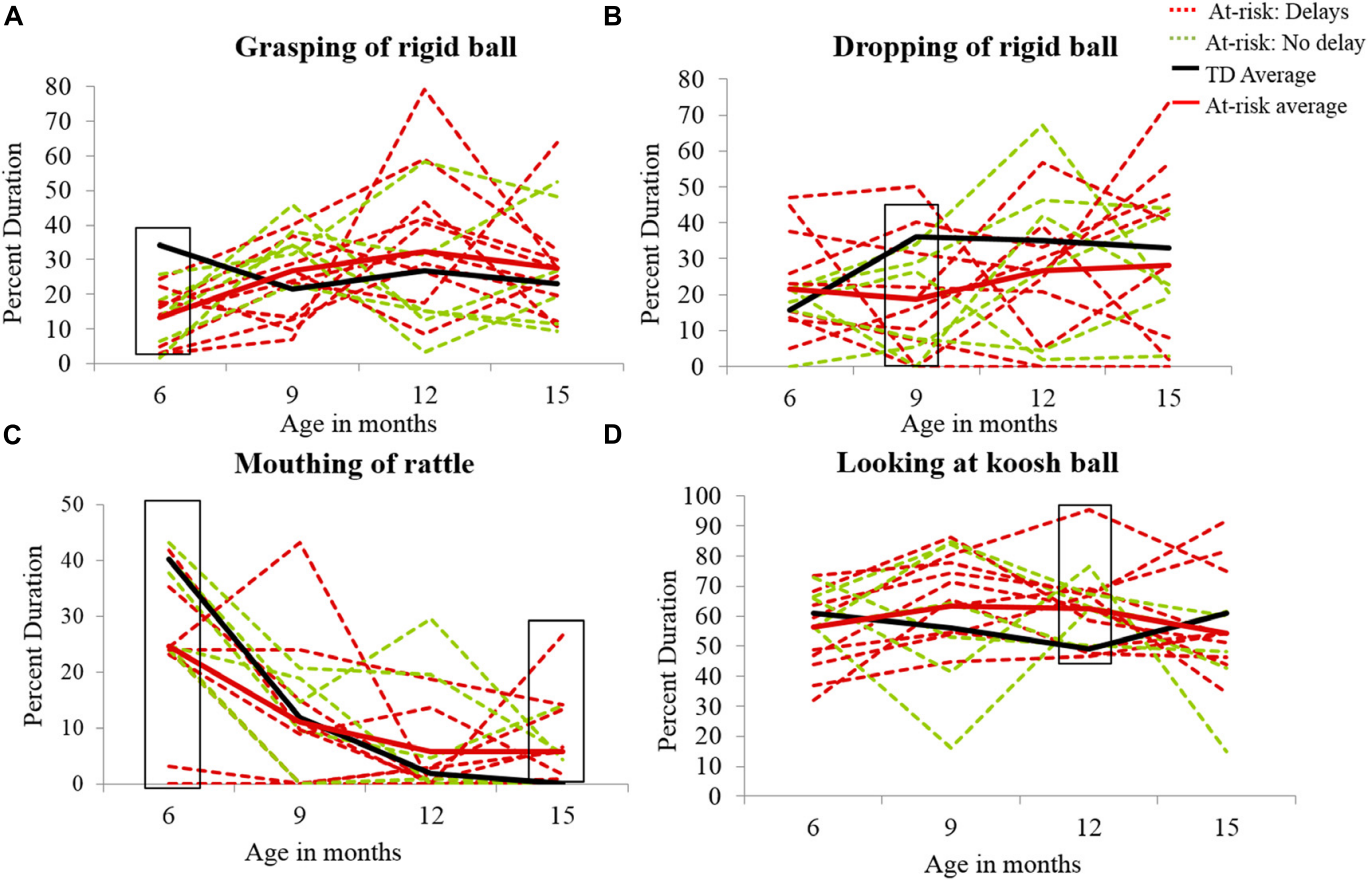
**Individual data for grasping of the rigid ball **(A)**, dropping of the rigid ball **(B)**, mouthing of the rattle **(C)**, and looking at the koosh ball **(D)** in at-risk infants.** The solid black line represents the average of the typically developing group and the solid red line represents the average of the at-risk group. The red dotted lines represent the 10 at-risk infants with future delays/ASD diagnosis, and the green dotted lines represent the at-risk infants without any future delays. The black boxes highlight the group differences with the majority of the at-risk infants performing worse than the typically developing group average.

In summary, early group differences observed included less grasping of the rigid ball, less mouthing of the rattle, greater looking at the rattle, and greater dropping of all three objects in at-risk infants compared to TD infants. The only mid group differences observed were lower levels of purposeful dropping at 9 and 12 months and greater looking at the koosh ball at 12 months in at-risk infants compared to the TD group. Lastly, in terms of late group differences, at-risk infants demonstrated persistent mouthing of the rattle and rigid ball compared to TD infants. In terms of individual data, 10–16 of the 16 at-risk infants always performed poorly compared to the TD group’s average values (see **Figures 7A–D**).

In terms of developmental changes, early changes for TD infants included reduced grasping with concurrent increases in purposeful dropping of multiple objects. In contrast, at-risk infants increased grasping early on and showed delays in the onset of purposeful dropping behaviors. Both TD and at-risk infants showed an early decrease in mouthing of objects. Mid changes for TD and at-risk infants included increased grasping of multiple objects. TD infants also continued to show a reduction in mouthing behaviors with the rattle, although similar changes were not observed in at-risk infants. At-risk infants began to develop purposeful dropping behaviors between 12 and 15 months. Late changes for TD infants included further reduction in mouthing behaviors and an increase in looking at the koosh ball. At-risk infants did not show any developmental changes in exploratory behaviors from 12 to 15 months. In terms of individual data, 12–16 out of the 16 TD infants and 12–15 out of the 16 at-risk infants followed their respective group trends.

### Individual Data for At-Risk Infants

Individual data from the at-risk infants are compared to the at-risk and TD group averages in **Figures 7A–D**. The 10 at-risk infants with future delays/ASD diagnosis have been highlighted in the figures as red dotted lines, the at-risk group’s average is a red solid line, and the TD group average is a black solid line. As discussed in the section “Group Differences and Differences in Development of Object Exploration in TD and At-Risk Infants,” at-risk infants showed poor grasping of the rigid ball at 6 months compared to the TD group average (see **Figure 7A**). In terms of dropping, the majority of the at-risk infants showed less dropping of the rigid ball than the TD group average at 9 months (see **Figure 7B**). In terms of mouthing, the majority of the at-risk infants showed less mouthing of the rattle at 6 months and persistently greater mouthing at 15 months than the TD group average (see in **Figure 7C**). Lastly, most of the at-risk infants showed greater looking at the koosh ball at 12 months compared to the TD group average. Moreover, looking periods appeared to be consistently higher in the at-risk infants across visits compared to the TD group average (see **Figure 7D**). It should be noted that the majority of the at-risk infants including at-risk infants without delays performed poorly compared to the TD average.

## Discussion

### Summary of Results

To our knowledge, this is the first study to longitudinally compare the developmental changes in visual, oral, and manual exploration using three different objects between TD and at-risk infants from 6 to 15 months of age. Both groups adapted their exploration to the unique properties of objects by demonstrating greater grasping and mouthing of the easily-graspable rattle, greater dropping of the sounding rigid ball, and greater looking at the novel koosh ball compared to the other objects (see section Object-Based Differences in Object Exploratory Behaviors in TD and At-Risk Infants and **Figures 2A–D**).

In terms of group differences in grasping, at-risk infants showed deficient grasping of the rigid ball at 6 months (see **Figures 3–6**). In addition, they showed deficient functional dropping of objects at 9 and 12 months compared to TD infants. In terms of mouthing, at-risk infants showed reduced mouthing of the rattle at 6 months but demonstrated persistent mouthing of the rattle and rigid ball at 15 months compared to TD infants. Lastly, at-risk infants showed greater looking at the rattle at 6 months as well as at the koosh ball at 12 months compared to TD infants.

In terms of developmental changes, we examined early (between 6 and 9 months), mid (between 9 and 12 months), and late (between 9 and 15 months) changes in object exploration of TD and at-risk infants (see section Group Differences and Differences in Development of Object Exploration in TD and At-Risk Infants and **Figures 3–6**). In terms of early changes for grasping and dropping, TD infants showed reduced grasping of the rigid ball with a concurrent increase in dropping. In contrast, at-risk infants showed increased grasping of the rattle and rigid ball with no onset of dropping. Mid changes for the TD and at-risk infants included increased grasping of various objects. In terms of late changes at-risk infants increased dropping of objects from 12 to 15 months. In terms of mouthing, TD infants showed high levels of mouthing early on but reduced mouthing behaviors over development whereas at-risk infants showed lower levels of mouthing early on with persistent mouthing at 15 months. Looking patterns did not change with development for both groups except for some increase in exploratory looking at the koosh ball in TD infants between 12 and 15 months.

### Object-Based Differences in Exploration

In the current study, infants were presented with three perceptually distinct objects that varied in terms of their shapes, sizes, and textures. Infants demonstrated greater grasping and mouthing of the rattle, greater dropping of the rigid ball, and greater looking at the koosh ball. These object-based differences in exploratory strategies could be due to salient differences in properties and affordances of objects as well as infants’ prior experiences with similar objects. For example, in terms of object properties, the cylindrical rattle allowed for a relatively easy hook grasp compared to the circular rigid ball that required a larger bimanual palmar grasp or the koosh ball that required more advanced coordination using a multi-digit pincer grip. Previous studies have also demonstrated variations in infants’ grasping patterns based on object structure, such as bimanual palmar grasps for larger objects and pincer grips for smaller and softer objects (Newell et al., 1989, 1993; Newman et al., 2001; Barrett et al., 2008). Similarly, infants in both groups seemed to have perceived specific object affordances and adapted their actions accordingly. Other studies have also shown that infants between 6 and 12 months typically perceive object affordances such as shaking, banging, and dropping (Ruff, 1986; Loucks and Sommerville, 2013). Along these lines, the rattle might have afforded grasping and shaking to produce a sound and the sounding rigid ball might have afforded throwing or dropping. Moreover, since mouthing behaviors are dependent on infants’ ability to grasp objects (Whyte et al., 1994), infants might have demonstrated greater mouthing and grasping of the rattle compared to other objects. Additionally, the narrow, cylindrical structure of the rattle makes it relatively easier to mouth compared to the wide and circular rigid ball or the filamentous koosh ball. Lastly, since the koosh ball is a relatively novel toy that is typically not a part of infants’ natural environment, it might have evoked greater visual fixation in both groups. In fact, early on, infants hesitated to grasp the koosh ball as they were unsure of its affordances and instead looked at it longer. Overall, there were several interesting object-based differences in exploration observed in both groups.

### Group Differences and Developmental Changes in At-Risk and TD Infants

In terms of group differences for grasping, at-risk infants showed less grasping of the rigid ball compared to TD infants at 6 months (see **Figure 3B**). Along these lines, grasping delays have been reported in AU sibs at 6 months of age within an object exploration task as well as on a standardized motor assessment, the fine motor sub-test of the Mullen Scales of Early Learning (Libertus et al., 2014). Grasping delays in at-risk infants in our study could be attributed to specific object properties of the rigid ball as well as to the postural and fine motor delays seen in at-risk infants. Specifically, the rigid ball used in our study was harder to grasp compared to the rattle and the koosh ball due to its large size, thereby requiring good bimanual control. Moreover, postural instability as well as poor fine motor control may have contributed to grasping delays in at-risk infants (Teitelbaum et al., 1998; Landa and Garrett-Mayer, 2006; Ozonoff et al., 2008b; Bhat et al., 2011; Nickel et al., 2013). For example, some of the early gross motor delays in at-risk infants include postural asymmetries as well as delayed acquisition of postures such as rolling, sitting, crawling, and walking (Teitelbaum et al., 1998; Ozonoff et al., 2008b; Nickel et al., 2013). Postural instability can lead an unstable base of support, which in turn can impair infants’ reaching and fine motor skills (Spencer et al., 2000). Moreover, infants who later developed ASD demonstrated poor fine manual control including delays in the onset of grasping, reaching, and pointing skills (Landa and Garett-Mayer, 2006; Gernsbacher et al., 2008). Overall, poor gross and fine motor control can significantly impair manual exploration skills of infants at-risk for ASD.

In terms of developmental changes in grasping, at-risk infants increased grasping of the rigid ball and rattle from 6 to 9 months and of the koosh ball from 9 to 12 months, whereas TD infants increased grasping of the rattle between 9 and 12 months. Infants are known to improve their grasping abilities between 6 and 15 months of age with a transition from ulnar grasps to radial palmar grasps (Butterworth et al., 1997). Similarly, 12- to 14-month-old infants’ showed appropriate, anticipatory changes in grasp formation based on object shape and size compared to 5- to 6-month-old infants (Fagard, 2000; Barrett et al., 2008). Along the same lines, we observed that infants began to engage in more sophisticated forms of manual exploration including fingering, shaking, banging, and rotating objects that could have contributed to an increase in grasping from 9 to 15 months. Currently, we are coding for more refined and sophisticated forms of manual exploration in both groups of infants.

In terms of group differences for dropping, at-risk infants spent greater time dropping objects at 6 months of age but demonstrated lower levels of functional dropping at 9 and 12 months compared to TD infants (see **Figure 4**). The greater dropping at 6 months in at-risk infants may be due to their fine motor delays leading to difficulties in grasping objects and unintentional slips while attempting to grasp toys. The reduced dropping at 9 and 12 months in at-risk infants may be an early indicator of poor functional and object-appropriate play in at-risk infants. A few other studies have also shown delayed functional play in AU sibs and infants later diagnosed with ASD during the first year of life (Baranek et al., 2005; Ozonoff et al., 2008a). Specifically, AU sibs showed non-functional use of objects such as excessive spinning and rotating of toys at 12 months of age (Ozonoff et al., 2008a).

In terms of development trends in dropping, at-risk infants demonstrated delayed emergence of functional dropping behaviors compared to TD infants. Dropping behaviors typically emerge between 9 and 12 months and increase with development (Ruff, 1986; Ruff et al., 1992). In our study, we observed that several TD infants engaged in dropping behaviors early on due to the specific sounding properties of rigid objects and to initiate social games with caregivers. Infants were seated in a high chair and dropping toys on the floor or on the table produced sounds that infants found appealing. Infants also used such behaviors as an opportunity to initiate interactions with caregivers as they checked back with them after purposefully dropping toys. Therefore, we think that dropping behaviors in TD infants were a form of early functional play. Along these lines, other research also suggests that TD infants manipulate sounding objects more often compared to non-sounding objects within the first year, suggesting that infants recognize object properties and engage in functionally appropriate actions (Palmer, 1989).

In terms of group differences in oral exploration of objects, at-risk infants’ demonstrated reduced mouthing of the rattle at 6 months and excessive mouthing of the rattle and rigid ball at 15 months compared to TD infants (see **Figures 5A,B**). Note, that the koosh ball was the least mouthed object due to its novel texture/appearance. Early delays in mouthing could be a function of poor grasping abilities. There is evidence to suggest that early on, oral exploration of objects is closely related to the manual exploratory skills of infants (Whyte et al., 1994) with better grasping allowing for easier mouthing. Given the early grasping delays observed among at-risk infants, it was not surprising that they also engaged in less mouthing at 6 months compared to TD infants. Similar delays in early mouthing abilities of AU sibs have been observed at 6 months of age in other studies (Bhat et al., 2009; Koterba et al., 2012).

In terms of developmental changes in mouthing, TD infants reduced mouthing of the rattle from 6 to 9 and 9 to 15 months (see **Figure 5A**); such an early decrease in mouthing fits with what is known in the literature (Belsky and Most, 1981; Ruff, 1984; Rochat, 1989). Mouthing is a predominant form of exploration at 6 months of age and is known to reduce after the onset of more refined forms of manual exploration (Belsky and Most, 1981; Ruff, 1984; Rochat, 1989). At-risk infants showed an early reduction in mouthing, however, they failed to reduce mouthing from 9 to 15 months resulting in persistent mouthing at 15 months. Excessive mouthing of objects has also been reported in infants later diagnosed with ASD between 9 and 12 months of age (Baranek, 1999). This unusual persistence of oral exploration in at-risk infants could be due to infants seeking additional sources of sensory input by mouthing or chewing inedible objects (Dunn et al., 2002; Baranek et al., 2006; Tomchek and Dunn, 2007). Tomchek and Dunn (2007) reported that 95% of their study sample of children with ASD between 3 and 6 years had a sensory processing dysfunction including an over- or under-responsiveness to different sensations (Tomchek and Dunn, 2007).

Lastly, in terms of visual exploration, both TD and at-risk infants demonstrated greater looking at the novel koosh ball at 6 months suggesting that both groups were equally enamored by this unfamiliar object. However, at-risk infants additionally showed excessive looking at the rattle at 6 months and at the koosh ball at 12 months compared to TD infants (see **Figures 6A,C**). Moreover, individual data in **Figure 7D** show a general trend for excessive visual exploration of objects in at-risk infants compared to TD infants. It is worth emphasizing that at-risk infants demonstrated excessive visual exploration irrespective of the novelty of objects used. For example, they looked more even at the relatively familiar rattle. Various studies have reported unusual visual fixation on objects in AU sibs (Ozonoff et al., 2008a; Bhat et al., 2010; Koterba et al., 2012) and their inability to disengage visual attention during the first year of life (Zwaigenbaum et al., 2005). Such excessive object fixation in AU sibs usually co-occurred with reduced attention to social partners and could directly contribute to the delayed social development in infants who eventually develop autism (Maestro et al., 2002, 2005; Bhat et al., 2010; Chawarska et al., 2013).

In terms of developmental changes in visual exploration, both at-risk and TD infants showed no changes in looking patterns except for increased looking at the koosh ball in TD infants from 12 to 15 months (see **Figure 6C**). This could be due to the development of more refined forms of manual exploration in TD infants requiring focused attention at the koosh ball while manipulating it in sophisticated ways. Our findings fit with those of another study where infants showed no clear changes in looking duration from 7 to 12 months during a free play-based task involving presentation of a variety of objects with distinct properties (Ruff, 1986).

Taken together, our longitudinal study comparing object exploration skills in TD and at-risk infants revealed that group differences in object exploration are highly context-dependent; delays in exploratory behaviors in at-risk infants are evident at different time points in development for specific objects with distinct affordances. Our study suggested that TD infants showed several advances in their strategies for object exploration from 6 to 15 months of age as a result of improvements in fine motor control as well as improved perception of object affordances. At-risk infants showed similar but delayed developmental trajectories in exploratory behaviors. For example, at-risk infants demonstrated grasping delays as well as a delayed emergence of functional dropping behaviors. In addition, they showed a reverse developmental trend for oral exploration, i.e., reduced early mouthing and persistent mouthing at later ages.

### Implications for Early Diagnosis and Treatment

The current study is unique in its approach of longitudinally studying various forms of object exploration concurrently in the context of objects with varying affordances in TD and at-risk infants within the first 15 months of life. We observed significant group differences in object exploration skills of at-risk infants from 6 to 15 months. Importantly, our study adds to the current literature by suggesting that group differences in exploratory behaviors are highly context-dependent such that delays in specific exploratory strategies are observed for specific objects and/or at specific ages. This has important implications for early screening as well as planning of object-based interventions for at-risk infants. Specifically, caregivers and clinicians should observe object play of infants within natural and structured settings for identifying early signs of autism risk. The set of objects used during exploratory play will play a crucial role in uncovering delays/atypicalities in object exploration skills in at-risk infants at different ages. Specific red flags for atypical object exploration during the first half of the first year include reduced oral and manual object exploration as well as increased visual regard for objects and other non-social stimuli. During the second half of the first year, a lack of age-appropriate and object-appropriate functional play such as shaking of sounding objects, dropping of ball-like objects, and fingering of soft objects could be signs of increased risk. In addition, at-risk infants may show persistent mouthing and unusually greater oral hyposensitivity.

Reduced and atypical object exploration could impact various forms of development in at-risk infants. Specifically, object exploration abilities are directly related to the development of cognitive skills such as object knowledge (Caruso, 1993; Bourgeois et al., 2005), non-verbal, and verbal communication skills such as the use of gestures and words within a social context (Fagan and Iverson, 2007; Iverson and Wozniak, 2007), as well as social skills such as imitation, joint attention, and pretend play (Bruckner and Yoder, 2007; McDuffie et al., 2012). Promoting object interactions within a social context will enhance multisystem development of infants at-risk for developing ASD. The use of object-based interventions can advance social skills such as turn taking and shared attention with caregivers as well as non-verbal and verbal communication skills such as showing and pointing to objects and object labeling (McDuffie et al., 2012). Object-based interventions could be implemented as early as 3 months to improve specific motor skills such as grasping and reaching in TD infants as well as AU sibs (Needham et al., 2002; Lobo and Galloway, 2008). Libertus and Landa (2014) reported enhancements in grasping following two weeks of active training with sticky mittens to facilitate object exploration in infants at-risk for autism. However, the same study did not find strong correlations between improved grasping performance and social attention in infants at-risk for autism suggesting that there are significant social delays in this population which may need substantial training to impact social attention at a young age as well as in the future (Libertus and Landa, 2014). It would be important to further investigate caregiver-training approaches to effectively use triadic interactions between at-risk infants and their caregivers within object exploration contexts to facilitate social interactions. Such forms of socially embedded object play also termed joint attention interventions are often used in preschool-based early intervention settings for children with autism (Kasari et al., 2010). For example, during the second half of the first year, caregivers could consider offering periods of free exploration and problem solving, model appropriate functional actions on objects, as well as engage in object sharing and pretend play with their infants. Caregivers must carefully select multiple objects with varying affordances to model actions, including everyday tools for pretend play and toys that promote sharing and cooperative play. Overall, object play could be a useful tool for early identification and treatment of infants at-risk for ASD and must be incorporated within early identification and intervention scenarios.

## Study Limitations

One of the limitations of our study was the slightly diverse at-risk group with the inclusion of two preterm twins. Preterm infants are a known population at-risk for ASD (Limperopoulos et al., 2008) and the two preterm infants included in our study received an ASD diagnosis after their second birthday. Another study limitation was the loss of data due to delayed recruitment, illnesses, and scheduling conflicts as is expected in longitudinal studies. However, the majority of the data were retained across all ages for both groups and did not appear to affect the overall group trends. The blocked presentation of objects may have influenced infants’ exploratory behaviors; however, the object-based differences appear to be meaningful and specific to the affordances of objects. Lastly, we clearly need to replicate our study results using larger sample sizes.

## Conclusion

The goal of the present study was to compare the early development of object exploration skills in infants at-risk for ASD and TD infants during the first 15 months of age along with follow-up at 18 and 24 months. While none of the TD infants developed delays or diagnoses in the future, several at-risk infants had multiple developmental delays or an ASD diagnosis. Our results indicate that at-risk infants demonstrated clear delays or abnormalities in object exploration such as early delays in grasping and mouthing, excessive visual exploration, reduced or delayed functional exploration of objects, and persistent mouthing later in life. Our study offers evidence to support the use of object exploration as a paradigm for early identification of perceptuo-motor delays and as an intervention context to promote motor, cognitive, and social communication skills in infants at-risk for developing autism.

## Conflict of Interest Statement

The authors declare that the research was conducted in the absence of any commercial or financial relationships that could be construed as a potential conflict of interest.
